# Management strategies for acute cholecystitis in late pregnancy: a multicenter retrospective study

**DOI:** 10.1186/s12893-023-02257-3

**Published:** 2023-11-10

**Authors:** Wei Zhang, Huiming Yi, Ming Cai, Jian Zhang

**Affiliations:** 1 Department of Medical Ultrasound, Tongji Hospital Affiliated to Tongji Medical College of Huazhong University of Science and Technology, Wuhan, Hubei Province 430030 China; 2 Department of Hepatobiliary Pancreatic Surgery, Tongji Hospital Affiliated to Tongji Medical College of Huazhong University of Science and Technology, Wuhan, Hubei Province 430030 China

**Keywords:** Late pregnancy, Acute cholecystitis, Conservative treatment, Percutaneous transhepatic gallbladder drainage, Laparoscopic cholecystectomy

## Abstract

**Objective:**

This study aims to investigate the management strategies for acute cholecystitis in the third trimester of pregnancy by comparing the effectiveness of three different treatments.

**Methods:**

Clinical data of 102 patients with acute cholecystitis in third trimester of pregnancy admitted to three Tertiary Hospitals from January 2010 to June 2020 were collected and divided into 3 groups according to the primary treatment during their first hospitalization: Group A (surgical group; n = 11), Group B (percutaneous transhepatic gallbladder drainage (PTGD) group, n = 29) and Group C (conservative treatment group, n = 62). The length of stay, readmission rate, and preterm delivery rate of each group were analyzed retrospectively.

**Results:**

The average age of patients included in this study was 29 ± 2.16 years with an average gestational cycle of 35.26 ± 1.02 weeks. The readmission rates of patients in groups A, B, and C were 9.09%, 24.14%, and 58.06%; the preterm delivery rates were 9.09%, 3.45%, and 12.90%; and the length of stay was 4.02 ± 1.02 days, 12.53 ± 2.21 days, and 11.22 ± 2.09 days, respectively. The readmission rate was lower in group A than in groups B and C, the preterm delivery rate was lower in group B than in groups A and C, and the length of stay was shorter in group A than in groups B and C (all with statistically significant differences, P < 0.05).

**Conclusion:**

Patients with acute cholecystitis in late pregnancy need to be appropriately graded for severity and offered a sound treatment strategy after a thorough assessment of the condition while taking into account the willingness of the patients. For patients with mild severity, conservative treatment can be adopted; for patients with moderate or severe inflammation, PTGD can be performed first for symptom control, and wait till after delivery for surgery to be considered; and in some cases of critical condition and poor symptom control, surgical intervention should be promptly performed.

## Introduction

The incidence of acute abdomen in pregnant women is about 1 in 500, and about 1.2% of patients undergoing abdominal surgery are found to have an unplanned pregnancy on preoperative examination [1]. Therefore, the actual incidence of combined acute abdomen in pregnancy may be higher. Among them, the incidence of acute cholecystitis in pregnancy is one of the common acute abdominal conditions in pregnancy, with an incidence of 0.1-0.6%, second only to acute appendicitis, and mostly occurs in the middle and late stages of pregnancy [2]. Acute cholecystitis during pregnancy can be complicated by complications such as gallbladder suppuration, perforation, cholangitis, acute pancreatitis, etc. In severe cases, it can cause infectious shock and pose a threat to the life of the patient and the fetus [3]. Due to lack of awareness and psychological fear, most patients with acute abdomen during pregnancy would request conservative medication treatment after diagnosis of acute cholecystitis [4, 5]. But in 27-36% cases, the symptoms cannot be effectively relieved after conservative treatment, and prolonged abdominal pain and vomiting can cause intrauterine distress of the fetus, which may induce contractions and the risk of miscarriage and preterm delivery [6]. Patients in late pregnancy are at higher risk of preterm delivery due to persistent symptoms of acute cholecystitis as a result of their physical changes such as smaller abdominal space and diaphragm elevation, which are of even greater concern to clinicians [6].

Recently, some clinical reports have affirmed the safety and efficacy of emergency laparoscopic cholecystectomy (LC) for acute cholecystitis in pregnancy, which is particularly recommended in the second trimester [7]. However, few studies specifically addressed the clinical management of acute cholecystitis in late pregnancy. There is still controversy as to whether it’s beneficial to perform surgery for acute cholecystitis in late pregnancy and there is no consensus on which approach is better: conservative medication treatment, gallbladder aspiration and drainage, or emergency laparoscopic cholecystectomy [8]. Therefore, we conducted this multicenter retrospective study aiming to evaluate the effectiveness of different treatment modalities for patients with acute cholecystitis in late pregnancy.

## Methods

### Clinical data collection and organization

This study included 102 patients with acute cholecystitis in the third trimester of pregnancy who received treatment and follow-up at the Department of Biliary and Pancreatic Surgery, Tongji Hospital, Tongji Medical College, Huazhong University of Science and Technology, the Department of Hepatobiliary and Pancreatic Surgery, Second People’s Hospital, Anhui Province, and the Department of Hepatobiliary and Pancreatic Surgery, First Hospital, Hainan Medical College, from January 2010 to June 2020. All methods of this study were carried out in accordance with relevant guidelines and regulations, which were approved by the Ethics Committee of the Tongji Hospital Affiliated to Tongji Medical College of Huazhong University of Science and Technology. We recognize that the number of patients (102) over a 10-year period might seem limited. However, it is important to consider the rarity of acute cholecystitis occurrence specifically in the third trimester of pregnancy. Our stringent inclusion and exclusion criteria were designed to ensure the purity and reliability of our data, and in doing so, they might have inadvertently limited the number of eligible patients. Moreover, given the potential risks associated with surgical interventions during pregnancy, many cases may have been managed conservatively without meeting our criteria for inclusion.

The inclusion criteria include: pregnant women in late pregnancy (28–40 weeks); meeting the diagnostic criteria for acute cholecystitis (TG18 guidelines [9]); for those with multiple hospitalizations, only the first hospitalization records were taken as the primary data for statistical analysis, and the treatment records of subsequent recurrent hospitalizations were included as follow-up data. Exclusion criteria include: those with previous underlying diseases; those who underwent cholecystectomy and cesarean section at the same time; those with gallbladder cancer on postoperative pathological examination; those with combined common bile duct stones, and/or acute cholangitis, and/or acute pancreatitis; those with incomplete clinical data; and those who lost contact during follow-up.

Patients were grouped according to their primary treatment modality: those who underwent LC during their first hospitalization were group A (surgical group; n = 11); those who underwent percutaneous transhepatic gallbladder drainage (PTGD) for symptom control were group B (PTGD group, n = 29); and those who were treated conservatively with medication alone without surgery or PTGD were group C (conservative group; n = 62). Clinical data of the patients including age, gestational week, gestational number, clinical symptoms, physical examination, laboratory findings, imaging data, diagnosis, main treatment modality, maternal and infant complications, readmission, preterm delivery, and length of hospitalization were collected. Among them, the length of stay in group A was defined as the time from admission to discharge after LC. The length of stay for patients in Groups B and C was calculated as the sum of the length of stay for each hospitalization including from the first admission to discharge after delivery (for those who did not have LC after delivery) or discharge after postpartum LC (for those who had LC after delivery). Follow-up was continued for six months from the last discharge.

Given the ten-year duration of this study, it might appear that the number of patients is modest. However, the rarity of acute cholecystitis in the third trimester of pregnancy combined with our stringent criteria ensures the accuracy and reliability of our data but could also explain the limited number of qualifying participants. Additionally, considering the potential complications of surgical procedures during pregnancy, many cases were likely managed more conservatively, further reducing the number of eligible patients.

### Main treatment modalities

Patients and their families were fully consulted before receiving all treatments in this study, and all signed informed consent forms. During the first admission: 62 patients chose conservative medication treatment at the beginning, among which 2 patients underwent LC after insignificant symptom relief, while 5 patients chose PTGD for the same reason; 29 patients adopted PTGD, one of whom still had significant abdominal pain and fever after PTGD and was referred for LC surgery; and 11 patients chose to receive emergency LC directly. All patients who underwent LC were not referred to laparotomy.

**Fig. 1:**
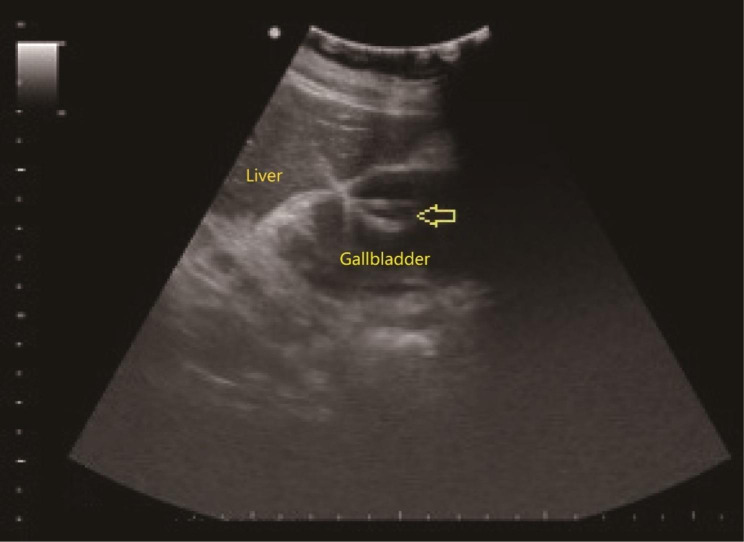
Image of ultrasound guided percutaneous transhepatic gallbladder drainage. An 8 F pigtail drainage catheter was placed in the gallbladder cavity (arrow)

The conservative treatment regimen mentioned above mainly includes diet control, appropriate fluid supplementation, correction of water-electrolyte disorders, anti-infection treatment (broad-spectrum cephalosporin antibiotics represented by cefoperazone), antispasmodic and analgesic treatment and contraction suppression treatment. PTGD is performed using a two-step approach in which: the ultrasound is positioned near the neck of the gallbladder, a 2-cm width of liver tissue was reserved along the puncture path; first, the needle is inserted into the gallbladder along a predetermined route with an 18G puncture needle under real-time ultrasound monitoring; then a guidewire is delivered and an 8 F pigtail drainage catheter is placed along the guidewire; the ultrasound confirms that the catheter is located inside the gallbladder, the pigtail drainage catheter lead is tightened and secured at the caudal end, and sutured to avoid dislodgement of the catheter (Fig. 1). The LC procedure was performed following the conventional approach, with the following points to be noted: adopt a head-high, foot-low, left lateral position, and do not change the body position too quickly during the procedure; use the Hasson method of opening instead of direct puncture with the Veress needle to establish the pneumoperitoneum; the first establishment is the Troca under the right costal margin instead of the umbilical port; consider ultrasound guidance if the pneumoperitoneum is difficult to establish; control the pneumoperitoneum pressure at 10–12 mmHg during the procedure and monitor with a CO_2_ analyzer.

Subsequent treatment: All 11 LC operations were successful, with no intraoperative need for conversion to laparotomy, and one patient was readmitted for post-cholecystectomy syndrome. Of the 29 patients who underwent PTCD, 7 had a recurrence of acute cholecystitis before (4 patients) or after (3 patients) delivery. Of the 62 patients who received conservative treatment, 36 had a recurrence of acute cholecystitis before (27 patients) or after (9 patients) delivery. All of the above patients with recurrent acute cholecystitis before delivery were treated again with anti-infective therapy followed by the remission of their condition and did not receive LC or other invasive procedures prior to delivery. Of all 91 patients who received conservative + PTCD treatment, 73 underwent LC surgery 3–6 months after giving birth.

After the first hospitalization for medical treatment in 102 patients in this study, the overall prognosis was assessed according to the incidence of maternal and infant complications, the incidence of preterm delivery, the readmission rate and the length of stay.

### Statistical analysis

SPSS 20.0 statistical software was applied, and normally distributed measurement data were expressed as —χ±s, and t-test was used for comparison between groups. The measurement data with skewed distribution were expressed as M (IQR). The χ2 test or Fisher’s exact probability method was used for count data. P < 0.05 was considered statistically significant.

## Results

### General information

A total of 102 patients with acute cholecystitis in late pregnancy (third trimester), with a mean age of 29 ± 2.16 years, a mean gestational cycle of 35.26 ± 1.02 weeks at first admission were included in this study. No statistical differences in terms of the mean age and gestational cycle existed among groups. (Table 1)

**Table 1:** Clinical data of patients with acute cholecystitis in late pregnancy

	Group A (n = 11)	Group B (n = 29)	Group C (n = 62)
**Age (years)**	29.47 ± 5.16	29.12 ± 3.16	28.92 ± 7.44
**Gestational cycle**	35.26 ± 1.22	34.75 ± 2.54	35.45 ± 1.94
**Symptoms and signs**			
** Right upper abdominal pain n (%)**	8 (72.72%)	20 (68.96%)	44 (70.97%)
** Fever n (%)**	2 (18.18%)	6 (20.69%)	1 (1.61%)
** Murphy positive n (%)**	6 (54.55%)	17 (58.62%)	3 (4.83%)
**Laboratory tests**			
WBC (*109/L)	16.54 ± 5.18	15.24 ± 3.18	11.77 ± 2.89
** ALT (IU/L)**	90.26 ± 4.33	86.12 ± 5.42	56.88 ± 2.89
** AST (IU/L)**	87.46 ± 7.52	88.33 ± 6.21	55.10 ± 1.99
** Total bilirubin (umol/L)**	15.86 ± 2.17	15.22 ± 1.97	15.16 ± 2.66
** Serum amylase**	42 ± 3.56	42 ± 4.55	43 ± 4.00
**Ultrasound examination**			
** Gallbladder size (mean, cm×cm)**	10.33 × 4.37	9.95 × 5.76	6.89 × 3.15
** Gallbladder wall thickness (mm)**	5.12 ± 0.33	4.85 ± 1.25	4.43 ± 2.25
**Grade n(I/II/III)**	1/7/3	1/25/3	51/10/1
**Length of hospitalization (days)**	4.02 ± 1.02	12.53 ± 2.21	11.22 ± 2.09
**Readmission rate n (%)**	1 (9.09%)	7 (24.14%)	36 (58.06%)
**Preterm birth rate n (%)**	1 (9.09%)	1 (3.45%)	8 (12.90%)

### Clinical manifestations, laboratory tests, imaging data and severity scores

Among the 102 patients with acute cholecystitis, 72 cases (72/102, 70.59%) presented with right upper abdominal pain, 9 cases (9/102, 8.82%) presented with fever, 26 cases (26/102, 25.49%) showed positive Murphy’s sign, and there was a higher percentage of patients in groups A and B presented with fever and positive Murphy’s sign than in group C (P < 0.05). There were no statistical differences between groups regarding other manifestations with diagnostic value.

All 102 patients received blood cell count, liver function, and amylase tests. The WBC, ALT and AST levels in groups A and B were higher than those in group C. The details are as follows: WBC: 16.54 ± 5.18*10^9^/L in group A, 15.24 ± 3.18*10^9^/L in group B, 11.77 ± 2.89*10^9^/L in group C, with both P_A-C_ and P_B-C_ <0.05; ALT: 90.26 ± 4.33 IU/L in group A, 86.12 ± 5.42 IU/L in group B, 56.88 ± 2.89 IU/L in group C, with both P_A-C_ and P_B-C_<0.05; AST: 87.46 ± 7.52 IU/L in group A, 88.33 ± 6.21 IU/L in group B, 55.10 ± 1.99 IU/L in group C, with both P_A-C_ and P_B-C_<0.05. There were no statistical differences in amylase, total bilirubin and GGT levels among groups.

**Fig. 2:**
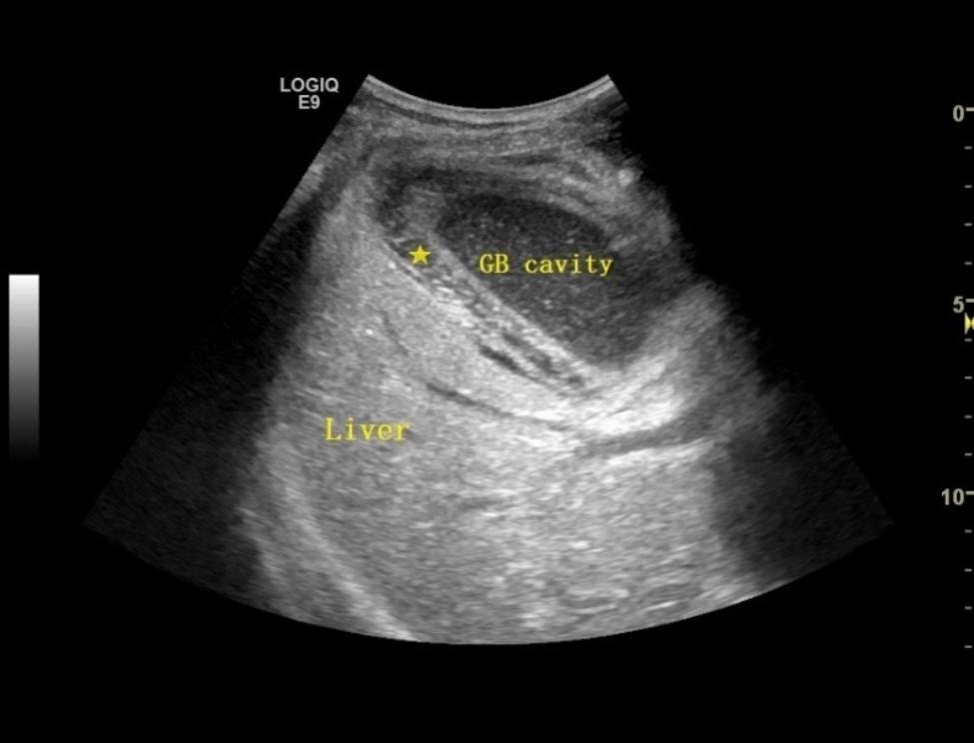
Ultrasound image of acute cholecystitis demonstrating thickened gallbladder wall (star) and dotted echogenicity in the gallbladder cavity. GB, gallbladder

According to Ultrasound examination focused on gallbladder size and gallbladder wall thickness (Fig. 2), the thickness of the gallbladder wall was 5.12 ± 0.33 mm, 4.85 ± 1.25 mm, and 4.43 ± 2.25 mm in group A, B, and C, respectively. And patients in groups A and B had larger gallbladder volumes and thicker gallbladder walls compared with patients in group C (P_A-C_, P_B-C_ <0.05, P_A-B_>0.05).

Combined with the above-mentioned examination results, according to the TG18 guidelines [9], for the severity of acute cholecystitis of patients at the time of first hospital admission, the numbers of Grade I/II/III patients in each group are as follows: 1/7/3 in group A, 1/25/3 in group B, and 51/10/1 in group C (with statistical differences between groups, P_A-C_, P_A-B,_ and P_B-C_ all less than 0.05), which shows a greater proportion of grade II and III patients in the surgery and PTGD groups.

### Follow-up and prognosis

For all cases in this study, the readmission rates in each group were 9.09% (1/11) in group A, 24.14% (7/29) in group B, and 58.06% (36/62) in group C. There were statistical differences between the groups (PA-C, PA-B, and PB-C were less than 0.05).Preterm delivery (delivery before 37 weeks) occurred in 1 of 11 patients (1/11, 9.09%) who underwent LC in group A; in 1 of 29 patients (1/29, 3.45%) who underwent PTGD in group B; and in 8 of 62 patients (8/62, 12.90%) who received conservative drug treatment in group C. Compared with groups A and C, patients in group B had a lower preterm delivery rate (P < 0.05). The mean length of stay in each group was 4.02 ± 1.02 days in group A, 12.53 ± 2.21 days in group B, and 11.22 ± 2.09 days in group C. The mean length of stay in group A was shorter than that in groups B and C (P < 0.05).

Although some pregnant women had preterm delivery and rehospitalization, fortunately no serious maternal and infant complications occurred, and no maternal or fetal death occurred.

## Discussion

In evaluating the choices of treatment modalities made by patients and their families, it’s pivotal to interpret them in the context of the varying clinical presentations. As our results indicate, there is a clear distinction in the severity of acute cholecystitis among the groups: patients in groups A and B exhibited more severe clinical symptoms and pathological markers, which correlates with the decision to pursue surgical intervention or percutaneous drainage. It’s essential to highlight the interplay between patient choice and clinical advisement. While the choice remains with the patient and family after a thorough consultation, the recommendations made by clinicians are anchored in the patient’s clinical condition. The apparent skew in treatment modalities, with those in more severe conditions leaning towards surgery or PTGD, likely reflects the weight of clinical recommendation in influencing patient decisions. Furthermore, it’s paramount to consider the natural apprehension surrounding surgical interventions, especially during pregnancy. A patient with less severe symptoms, even when presented with the option of surgery, might lean towards conservative treatment out of concern for potential complications that could affect the pregnancy or the fetus. This proclivity can explain why patients in group C, who generally had milder symptoms, overwhelmingly chose conservative treatment. Another key aspect to discuss is the pathophysiology of acute cholecystitis in pregnancy. Hormonal changes during pregnancy, primarily progesterone, can affect gallbladder motility, potentially leading to cholestasis and subsequent stone formation or inflammation. Given this predisposition, it’s conceivable that some patients might experience acute episodes that are more amenable to conservative management, while others, possibly due to pre-existing biliary issues, might suffer from more aggressive manifestations necessitating surgical intervention or drainage. In conclusion, the differential clinical severities among the groups, when juxtaposed with the treatment modalities chosen, underscore the critical role of clinician advice, patient apprehensions, and inherent pathophysiological variations in acute cholecystitis during pregnancy.

Due to physiological changes and anatomical changes resulting from enlarged uterus and upward shift diaphragm, the management of acute cholecystitis in late pregnancy is a difficult issue in clinic, which involves both the mother and the fetus [2, 10]. Both surgery and general anesthesia have the potential to adversely affect the mother and fetus, and the use of carbon dioxide to establish a pneumoperitoneum during LC may lead to fetal hypoxia and increased rates of preterm delivery and fetal mortality [11]. In addition, studies found that the majority of patients (73-87%) were able to control their symptoms effectively with conservative treatment [4, 8]. Therefore, conventional strategy recommends that conservative treatment be given priority and surgical intervention only in cases where the patient is severely ill [6]. In our study of 102 patients, conservative treatment was adopted in 69 cases initially, of which 7 cases were referred to LC or PTGD. 82.26% (51/62) of patients in group C were Grade I patients, and the overall efficiency of conservative treatment was 88.71% (55/62). Therefore, this study supports the preferential use of conservative treatment in patients with grade I acute cholecystitis in late pregnancy. However, in this study, 58.06% (36/62) patients treated conservatively were rehospitalized before delivery due to recurrence of acute cholecystitis, and 12.90% (8/62) had preterm delivery, maintaining the overall rehospitalization and preterm delivery rates at a relatively high level. Therefore, the conservative treatment still carries a greater risk for both mother and baby, since recurrent inflammatory episodes are also important influencing factors for preterm delivery and maternal and infant complications.

For pregnant women whose symptoms are difficult to be relieved by conservative treatment, PTGD treatment can be performed prior to postpartum, which is relatively less traumatic and has less impact on the mother and fetus, in line with the principle of damage control management [12, 13]. In this study, 29 patients chose to adopt this strategy with a high efficiency of 96.55% (28/29), and only one patient in this group underwent emergency LC due to persistent uncontrolled symptoms. Among 29 patients who underwent PTGD in this study, 24.14% (7/29) were readmitted for recurrence of acute cholecystitis and 3.45% (1/29) had preterm delivery. The damage control treatment strategy is particularly superior to conservative treatment and LC in terms of lower rate of preterm delivery, since both recurrent inflammatory episodes after conservative treatment and surgical irritation of the abdominal cavity during LC may increase the chance of preterm delivery. In addition, 86.20% (25/29) of patients in group B were grade II acute cholecystitis. Furthermore, there were three patients with grade III acute cholecystitis adopting PTGD, two of which achieved good symptom control and one of which switched to LC with intraoperative confirmation of gallbladder gangrene. PTGD can prevent the progression of the disease through adequate drainage and relief of intra-gallbladder hypertension, avoid local ischemia, perforation and necrosis induced by vascular occlusion in the gallbladder wall [14]. Moreover, the drug sensitivity test derived from the culture of the extracted bile can be used to optimize antibiotic treatments, leading to a better clinical outcome [15]. Nevertheless, in patients with gangrene or even perforation of the gallbladder wall, PTGD is obviously not able to solve the underlying problem. Therefore, this study suggests that PTGD can be considered as a priority recommendation for patients with Grade II acute cholecystitis in late pregnancy. As for Grade III patients, PTGD may also be considered in the absence of gallbladder gangrene and perforation.

The gold standard for the treatment of acute cholecystitis is LC under general anesthesia [16], and the management strategies for different stages of acute cholecystitis in pregnancy are not quite the same compared with the conventional population [10, 11]. Currently, the safety and efficacy of LC for acute cholecystitis in mid-pregnancy has been demonstrated, and surgical treatment in early pregnancy is generally not recommended because it is a critical period for fetal development [7, 17]. However, the need for LC in patients with acute cholecystitis in late pregnancy is still controversial [1, 5, 6, 11]. In this study, 11 patients underwent LC surgery without serious maternal or fetal complications, and with one rehospitalization for post-cholecystectomy syndrome. The mean length of stay was shorter in the surgery group than in the conservative treatment and PTGD groups, but the preterm delivery rate was higher in the LC group at 9.09% (1/11) than in the PTGD group at 3.45% (1/29). These results reflected the safety and efficacy of LC surgery in patients with late pregnancy, but it may be inferior to PTGD treatment in controlling the preterm birth rate, which may be related to surgical stress. Therefore, this study suggests that LC should be applied in patients with acute cholecystitis in late pregnancy who are not suitable for conservative treatment with PTGD.

The diagnosis of advanced acute cholecystitis in pregnancy requires adequate clinical data collection and reasonable severity grading, In all cases in this study, the fever rate, Murphy’s sign positive rate, WBC, ALT, AST, and gallbladder wall thickness were higher in patients in the LC and PTGD groups than in the conservative treatment group and tended to be consistent with severity of the disease in each group. These results suggest that factors such as fever, abdominal signs WBC value, liver function, and gallbladder wall thickness can be used to evaluate the severity of acute cholecystitis in late pregnancy and provide a rational reference for the selection of treatment plan.

The Grade classification is recommended to assess the condition adequately and combine it with the patient’s wishes before a reasonable treatment strategy can be given. For patients with milder disease (Grade I), conservative treatment is indicated; for patients with moderate inflammation (Grade II), a damage control strategy, i.e., PTGD followed by deferred surgery, is recommended; for patients with more complicated inflammation (Grade III), a cautious choice is needed if PTGD does not provide good symptom control, or if there is a high suspicion of gangrenous perforation of the gallbladder wall, LC is recommended.

## Conclusions

Patients with acute cholecystitis in late pregnancy need to be appropriately graded for severity and offered a sound treatment strategy after a thorough assessment of the condition while taking into account the willingness of the patients. For patients with mild severity, conservative treatment can be adopted; for patients with moderate or severe inflammation, PTGD can be performed first for symptom control, and wait till after delivery for surgery to be considered; and in some cases of critical condition and poor symptom control, surgical intervention should be promptly performed.

## Data Availability

The datasets used and/or analyzed during the present study are available from the corresponding author on reasonable request.
